# Lung Ultrasound Assessment of Focal and Non-focal Lung Morphology in Patients With Acute Respiratory Distress Syndrome

**DOI:** 10.3389/fphys.2021.730857

**Published:** 2021-09-14

**Authors:** Charalampos Pierrakos, Marry R. Smit, Luigi Pisani, Frederique Paulus, Marcus J. Schultz, Jean-Michel Constantin, Davide Chiumello, Francesco Mojoli, Silvia Mongodi, Lieuwe D. J. Bos

**Affiliations:** ^1^Department of Intensive Care, Amsterdam UMC, Location AMC, University of Amsterdam, Amsterdam, Netherlands; ^2^Department of Intensive Care, Brugmann University Hospital, Université Libre de Bruxelles, Brussels, Belgium; ^3^Department of Anesthesia and Intensive Care, Miulli Regional Hospital, Acquaviva delle Fonti, Italy; ^4^Mahidol Oxford Tropical Medicine Research Unit, Mahidol University, Bangkok, Thailand; ^5^Laboratory of Experimental Intensive Care and Anesthesiology, Amsterdam UMC, Location AMC, University of Amsterdam, Amsterdam, Netherlands; ^6^Nuffield Department of Medicine, University of Oxford, Oxford, United Kingdom; ^7^Department of Anaesthesiology and Critical Care, Pitié-Salpêtrière Hospital, Sorbonne University, Paris, France; ^8^Dipartimento di Emergenza Urgenza, SC Anestesia e Rianimazione, ASST Santi Paolo e Carlo, Milan, Italy; ^9^Centro di Ricerca Coordinata di Insufficienza Respiratoria, University of Milan, Milan, Italy; ^10^Anaesthesia and Intensive Care, San Matteo Hospital, Pavia, Italy; ^11^Department of Clinical, Surgical, Diagnostic and Pediatric Sciences, University of Pavia, Pavia, Italy; ^12^Department of Respiratory Medicine, Amsterdam UMC, Location AMC, University of Amsterdam, Amsterdam, Netherlands

**Keywords:** lung ultrasonography, phenotype, mechanical ventilation, ICU, ARDS

## Abstract

**Background:** The identification of phenotypes based on lung morphology can be helpful to better target mechanical ventilation of individual patients with acute respiratory distress syndrome (ARDS). We aimed to assess the accuracy of lung ultrasound (LUS) methods for classification of lung morphology in critically ill ARDS patients under mechanical ventilation.

**Methods:** This was a *post hoc* analysis on two prospective studies that performed LUS and chest computed tomography (CT) scanning at the same time. Expert panels from the two participating centers separately developed two LUS methods for classifying lung morphology based on LUS aeration scores from a 12-region exam (Amsterdam and Lombardy method). Moreover, a previously developed LUS method based on anterior LUS scores was tested (Piedmont method). Sensitivity and specificity of all three LUS methods was assessed in the cohort of the other center(s) by using CT as the gold standard for classification of lung morphology.

**Results:** The Amsterdam and Lombardy cohorts consisted of 32 and 19 ARDS patients, respectively. From these patients, 23 (45%) had focal lung morphology while others had non-focal lung morphology. The Amsterdam method could classify focal lung morphology with a sensitivity of 77% and a specificity of 100%, while the Lombardy method had a sensitivity and specificity of 100 and 61%. The Piedmont method had a sensitivity and specificity of 91 and 75% when tested on both cohorts. With both the Amsterdam and Lombardy method, most patients could be classified based on the anterior regions alone.

**Conclusion:** LUS-based methods can accurately classify lung morphology in invasively ventilated ARDS patients compared to gold standard chest CT. The anterior LUS regions showed to be the most discriminant between focal and non-focal lung morphology, although accuracy increased moderately when lateral and posterior LUS regions were integrated in the method.

## Introduction

Acute respiratory distress syndrome (ARDS) is a frequent cause of hypoxemic respiratory failure and is characterized by protein rich pulmonary edema (Matthay et al., 2019). Diagnosis is based on a set of clinical and radiological criteria (Ferguson et al., 2012; Ranieri et al., 2012), resulting in remarkable physiological, radiological, and biological heterogeneity (Bos et al., 2018, 2021; Sinha and Calfee, 2019; Matthay et al., 2020). The notion that there is no “typical” ARDS may explain the failure of large clinical trials to demonstrate beneficial effects of unselective application of therapeutic interventions (Bos et al., 2021).

The identification of ARDS phenotypes can be helpful to better target treatment of individual patients with ARDS (Matthay et al., 2020). Lung imaging with computed tomography (CT) has been used to differentiate two distinct phenotypes of ARDS based on lung morphology. Lungs with diffuse and patchy loss of aeration (non-focal phenotype) generally respond well to recruitment while lungs with predominant dorso-inferior consolidations (focal phenotype) respond better to prone positioning (Constantin et al., 2010). Misclassification of these two different phenotypes results in misaligned ventilation strategies and is related to a substantial increase in mortality (Constantin et al., 2019). Therefore, it is pivotal to accurately recognize morphological phenotypes before a personalized strategy can be applied.

While CT scan remains the gold standard for lung assessment, it has several inherent limitations. It requires transportation to radiology department, which can be at high risk for critically ill patients, and requires moderate doses of radiation yielding it unsuitable as a monitoring tool. Furthermore, interpretation of morphology requires considerable expertise, which is pivotal in avoiding misclassification (Constantin et al., 2019). Chest X-rays are commonly performed in the intensive care unit (ICU), but recognition of focal and non-focal ARDS phenotypes remains challenging in these images (Constantin et al., 2019). Therefore, a bedside, simple and easily repeatable imaging tool would ideally provide useful information to manage ARDS patients in everyday practice.

Lung ultrasound (LUS) is a bed-side imaging technique that has been used to evaluate critically ill patients with acute respiratory failure (Mojoli et al., 2019). LUS has potential in both diagnosis and monitoring of ARDS and showed a good correlation with chest CT in estimating lung aeration (Riviello et al., 2016; Chiumello et al., 2018; Mongodi et al., 2019a; Pisani et al., 2019). A previously performed study showed promising results for LUS as a tool to classify lung morphology, but the percentage of patients with focal lung morphology was exceptionally low in this population and moreover the method lacks external validation (Costamagna et al., 2021). We aimed to assess the accuracy of LUS methods for classification of lung morphology in critically ill ARDS patients under mechanical ventilation. We hypothesized that LUS can reliably assess lung morphology compared to gold standard chest CT (Mongodi et al., 2019b).

## Materials and Methods

### Study Design and Ethical Concerns

We performed a *post hoc* analysis on two prospective studies that performed LUS and chest CT scanning at the same time. The Amsterdam cohort consisted of invasively ventilated patients included in prospective observational study performed in the ICU of the Amsterdam University Medical Centers, location “Academic Medical Center” (AMC), Amsterdam, Netherlands (Smit et al., 2021). The study protocol was approved by the institutional review board (IRB) of the AMC (2017_312#B201859). Patients in this study were analyzed if they fulfilled the Berlin criteria of ARDS (Ranieri et al., 2012). The Lombardy cohort consisted of patients from a study in invasively ventilated ARDS patients performed at the ICU of the Fondazione IRCCS Cà Granda Ospedale Maggiore Policlinico, Milan, Italy. This study was approved by the hospitals’ IRB (Chiumello et al., 2018).

### Definitions

**FIGURE 1:**
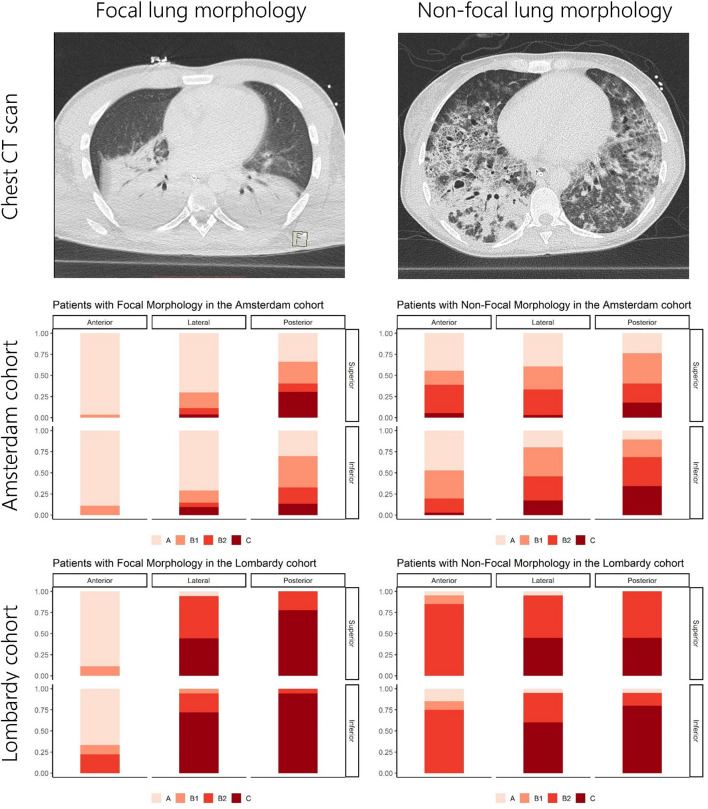
Overview of LUS patterns present per lung region for the Amsterdam and Lombardy cohorts. The upper images show examples of CT images from patients with focal and non-focal lung morphology. The middle and lower figures show the distribution of LUS patterns [A-pattern (score 0), B-patterns (scores 1 and 2), and C-patterns (score 3)] for the anterior, lateral, and posterior lung regions stratified for lung morphology as assessed by CT and cohort. LUS, lung ultrasound; CT, computed tomography.

Focal morphology was defined as isolated consolidations with an infero-dorsal dominance as assessed with CT. Non-focal morphology was defined as presence of diffuse or patchy opacifications, with or without dorsal consolidations (Figure 1 and Supplementary Figure 1).

### Lung Morphology Assessed With CT

Chest CT scans of both studies were evaluated and characterized as focal or non-focal by at least two investigators. In case of disagreement, the scans were discussed in a panel of at least three investigators until a consensus was reached. This classification was performed while blinded for the results of the LUS exam and were used as the reference standard in all subsequent analysis. Evaluation of LUS and CT images in the Amsterdam and Lombardy cohort was performed independently by researchers from the respected centers.

### LUS Examination

**FIGURE 2:**
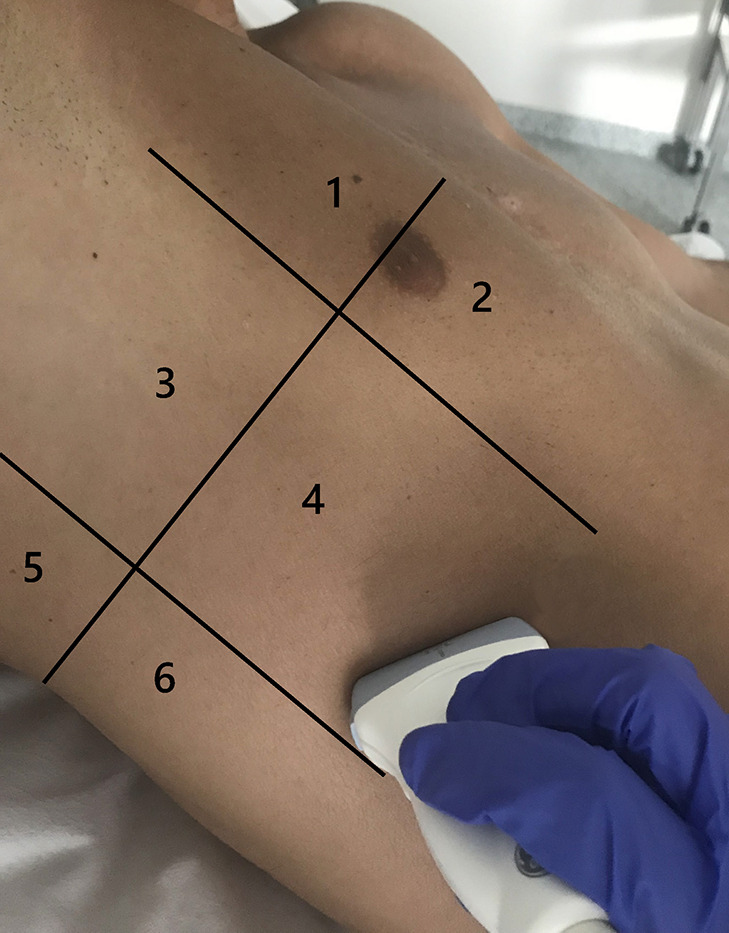
Lung regions scanned in a 12-region LUS exam shown for one hemithorax. LUS images were acquired using a linear transducer and a transversal approach. Zones 1 and 2 are anterior LUS regions, zones 3 and 4 are lateral LUS regions, and zones 5 and 6 are posterior LUS regions. LUS, lung ultrasound.

**FIGURE 3:**
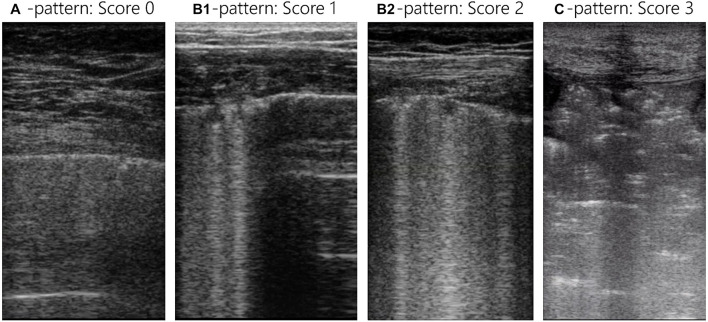
Lung ultrasound images for all LUS patterns and scores. Each LUS image was scored with the LUS aeration score: an “A-pattern” (i.e., repeating horizontal A-lines parallel to the pleural line) was scored “0,” a “B-pattern” (i.e., ≥3 vertical B-lines starting from the pleural line and reaching the bottom of the screen) was scored “1” if B-lines are well-spaced and cover ≤50% of the pleural line, and “2” if B-lines cover ≥50% of the pleural line, and a “C-pattern” (i.e., consolidation) was scored “3.” The global LUS score is the sum of all 12 lung regions and reaches from 0 to 36 and the anterior, lateral, and posterior LUS score are the sum of 4 lung regions and reach from 0 to 12. LUS, lung ultrasound.

In the Lombardy cohort, LUS was performed immediately before or after the CT examination with the same ventilator and using identical settings; the original protocol assessed patients at positive end-expiratory pressure (PEEP) of 5 and 15 cmH_2_O, but only the CT and LUS exams at a PEEP level of 5 cmH_2_O were analyzed for the present study (Chiumello et al., 2018). In the Amsterdam cohort, LUS was performed in the ICU just before transport to the CT scanner with the patient connected to the transport ventilator. The PEEP level in the Amsterdam cohort remained at the clinical PEEP level as set by the treating physician and was equal during LUS and CT. In both studies the LUS exam was performed using an identical 12-region protocol with patients in semi-recumbent position. Each hemithorax was divided into six regions: anterior, lateral, and posterior fields were identified by sternum, anterior, and posterior axillary lines; each field was further divided into superior and inferior regions (Figure 2). The regions were scanned with a transversal approach – i.e., the probe aligned with the intercostal space – to maximize lung exposition and minimize rib related shadowing; the scanning area was centered in the region of interest (Figure 3 and Supplementary Figure 2). LUS videos were stored and scored off-line by sonographers with extensive expertise in LUS blinded for the findings on the chest CT scan. A regional score was computed according to the visualized artifacts: (1) an “A-pattern” (i.e., repeating horizontal A-lines parallel to the pleural line, suggesting normal aeration) was scored “0,” (2) a “B-pattern” (i.e., three or more vertical B-lines starting from the pleural line and reaching the bottom of the screen, suggesting partial loss of aeration) was scored “1” if B-lines are well-spaced and cover ≤50% of the pleural line, and “2” if B-lines cover ≥50% of the pleural line, and (3) a “C-pattern” (i.e., consolidation, suggesting near-complete to complete loss of aeration) was scored “3” (Bouhemad et al., 2007; Brusasco et al., 2019; Mongodi et al., 2021; Figure 3). Examples of LUS clips are added as Supplementary Videos 1–6. Missing LUS images were complemented by the mean LUS aeration score of the other available LUS images in the concerning region (anterior, lateral, or posterior region). The global LUS aeration score was defined as the sum of LUS aeration scores from all 12 images; anterior, lateral, and posterior LUS scores were computed as the sum of anterior, lateral, and posterior regions, respectively.

### Derivation of the LUS-Based Method

**FIGURE 4:**
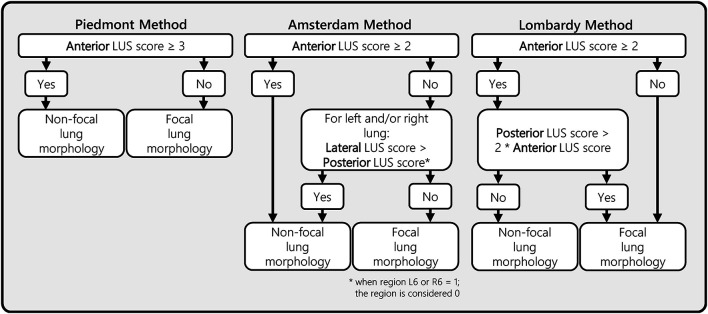
Ultrasound lung morphology assessment methods. This figure presents three LUS morphology assessment methods that were designed and/or evaluated in this study. All three methods classify focal or non-focal lung morphology based on LUS aeration scores from a 12-region LUS exam. The anterior, lateral, and posterior LUS scores were defined as the sum of the LUS aeration score in the four anterior, lateral, and posterior regions, respectively. The Piedmont method was previously proposed in a study from Costamagna et al. (2021). The Amsterdam and Lombardy method were developed for the purpose of this study by two expert panels from the corresponding regions. LUS, lung ultrasound.

Lung morphology assessment through LUS was assessed with three different methods. One previously published method by Costamagna et al. (2021) (Piedmont method) and two methods that were developed by expert panels in Amsterdam and Lombardy. The Piedmont method considered lung morphology as non-focal when patients had an anterior LUS score larger or equal than 3, and remaining patients as having focal lung morphology (Costamagna et al., 2021). The Amsterdam and Lombardy method were independently developed based on the LUS and CT data from the corresponding cohort (Amsterdam and Lombardy cohorts). Both of these methods were based on a stepwise approach starting with the evaluation of the anterior LUS score. In the second step, the posterior LUS score was either compared with the lateral LUS score (Amsterdam method) or with the anterior LUS score (Lombardy method) (Figure 4).

### Validation of the LUS-Based Methods

Performance of the three LUS methods was assessed by using the methods to classify lung morphology in the cohort of the other center(s). No further changes were allowed to the methods during the validation phase.

### Endpoints

The primary endpoint of the study was the sensitivity and specificity of the LUS-based methods (index test) for lung morphology based on the CT scan (reference test). The secondary endpoints were (1) the comparison of anterior, lateral, and posterior LUS scores, all stratified for focal and non-focal lung morphology, (2) comparison of the three LUS-based methods when applied to both cohorts combined, and (3) identification of best cut off point for the anterior LUS score in both cohorts combined.

### Statistical Analysis

Demographic and clinical variables were presented as percentages for categorical variables and as medians with interquartile ranges (IQRs) for continuous variables. Categorical variables were compared with the Chi-squared test and continuous variables were compared with the Mann–Whitney *U* test. Based on the lung morphology classifications of LUS and CT, contingency tables were generated to characterize the sensitivity and specificity of the method with respect to the reference standard. Sensitivity, specificity, disease prevalence, positive predictive value (PPV), and negative predictive value (NPV) as well as accuracy were calculated and expressed as percentages. Moreover, the F1-score and Matthews correlation coefficients were calculated. No formal power calculation was performed. Differences in classification accuracy between the LUS methods were assessed by comparing receiver operating characteristic (ROC) curves and calculating the categorical net-reclassification index (NRI) and integrated discrimination index (IDI) using R (R Development Core Team R, 2011) through the R-studio interface (Version 1.2.1335) using data of both the Amsterdam and Lombardy cohort.

## Results

### Patient Population

The Amsterdam and Lombardy cohort consisted of 32 and 19 patients, respectively. Patient characteristics are presented in Table 1. Fourteen patients (44%) had focal morphology in the Amsterdam cohort and nine (47%) in the Lombardy cohort (*p* = 0.84). LUS scores per region for both cohorts and a CT example of focal and non-focal lung morphology is presented in Figure 1. Patients in the Amsterdam cohort had a lower global LUS score compared to patients in the Lombardy cohort [13 (7–17) vs. 25 (23–29), *p* < 0.01]. The global LUS score was also lower in the Amsterdam cohort compared to the Lombardy cohort in patients with mild ARDS [11 (4–16) vs. 24 (20–24), *p* < 0.01] and moderate ARDS [15 (8–17) vs. 25 (23–29), *p* < 0.01]. Only one patient in the Amsterdam cohort had severe ARDS.

**TABLE 1:** Characteristics of the patients included in the Amsterdam and Lombardy cohorts examined with lung ultrasound and computed tomography.

Characteristic	Amsterdam cohort		Lombardy cohort	
	Focal ARDS *N* = 14	Non-focal ARDS *N* = 18	Focal ARDS *N* = 9	Non-focal ARDS *N* = 10
Age, median (IQR), years	57 (37–67)	59 (56–68)	59 (47–75)	55 (45–73)
Female, no. (%)	3 (21)	5 (28)	4 (44)	3 (30)
Duration of invasive ventilation before enrollment, median (IQR), days	4 (2–8)	4 (1–6)	2 (2–4)	5 (2–10)
ICU mortality, no. (%)	6 (43)	6 (33)	6 (67)	5 (71)^a^
Global LUS score	7 (2–9)	16 (12–18)	24 (22–25)	28 (25–31)
ARDS severity				
Mild, no. (%)	8 (57)	8 (44)	3 (33)	2 (20)
Moderate, no. (%)	5 (36)	10 (56)	5 (56)	6 (60)
Severe, no. (%)	1 (7)	0	1 (11)	2 (20)
Respiratory measures, median (IQR)				
PaO_2_ to FiO_2_ ratio	255 (135–289)	199 (138–233)	176 (113–225)	152 (103–180)
FiO_2_, %	50 (40–60)	60 (50–65)	50 (40–59)	50 (50–65)
Tidal volume, mL	503 (411–551)	433 (349–582)	450 (340–520)	450 (325–550)
Positive end-expiratory pressure, cm H_2_O	7 (5–8)	10 (8–12)	5 (5–5)	5 (5–5)
Respiratory rate, breaths/min	22 (16–25)	26 (17–35)	18 (16–25)	15 (10–16)

*ARDS, acute respiratory distress syndrome; IQR, inter-quartile range; LUS, lung ultrasound; ICU, intensive care unit; PaO_2_, partial pressure of oxygen; FiO_2_, fraction of inspired oxygen.*

*^*a*^Data available in 7 out of 10 patients.*

In the Amsterdam cohort, 46 out of 384 (12%) LUS images were missing due to chest tubes, subcutaneous emphysema or morbid obesity [median of 1 (0–2) regions per patient]. From the missing LUS images, 1, 12, and 33 images were missing in the anterior, lateral, and posterior region, respectively. The Lombardy cohort did not have missing LUS images. Additionally, the PEEP level was 8 (5–11) cmH_2_O in the Amsterdam cohort and 5 (5–5) cmH_2_O in the Lombardy cohort (*p* < 0.01).

### Diagnostic Performance

The diagnostic performance of the three methods for detecting focal morphology is presented in Table 2. The performance of the Piedmont method was moderate to good when tested on data of the Amsterdam and Lombardy cohort combined with a sensitivity of 91% and a specificity of 75%, for detecting focal lung morphology. The Amsterdam method had a good performance when tested on data of the Lombardy cohort with a sensitivity of 77% and specificity of 100% for the detection of focal lung morphology. The Lombardy method had a moderate performance when tested on data of the Amsterdam cohort with a sensitivity of 100% and a specificity of 61% for the detection of focal lung morphology.

**TABLE 2:**
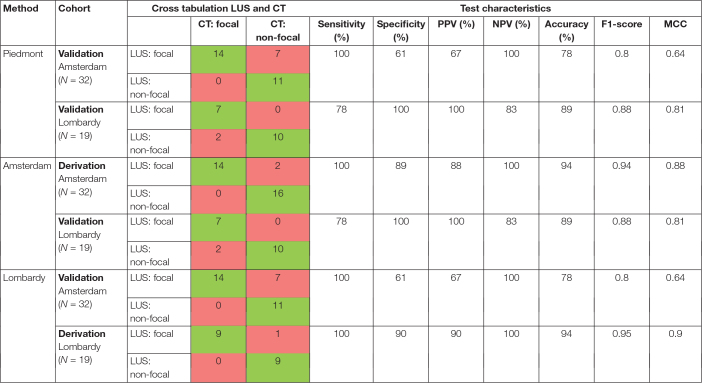
Distribution of examined patients according to their lung morphology determined with LUS-based method in comparison to CT findings.

*LUS, lung ultrasound; CT, computed tomography; PPV, positive predictive value; NPV, negative predictive value; MCC, Matthews correlation coefficient.*

*The green shade in the cross tabulation represents correctly classified patients and the red shade represents incorrectly classified patients.*

**FIGURE 5:**
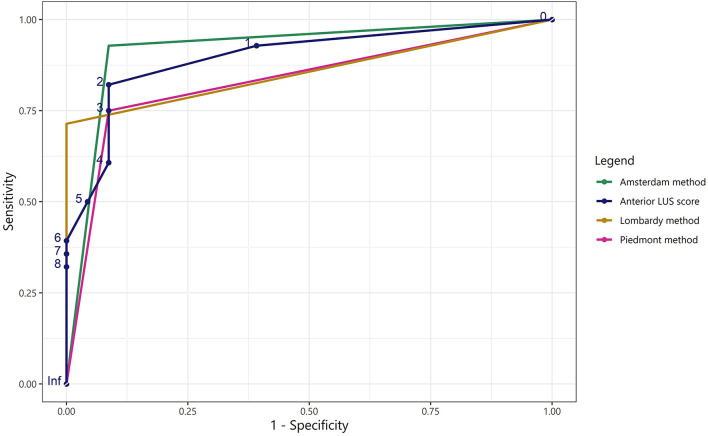
Receiver operating characteristic curves for the LUS methods and the anterior LUS score in predicting non-focal lung morphology. ROC curves for the Piedmont, Amsterdam, and Lombardy method and for the anterior LUS score regarding classification of non-focal lung morphology when applied to both the Amsterdam and Lombardy cohort. The area under the ROC curve was: 0.83 for the Piedmont method, 0.92 for the Amsterdam method, 0.86 for the Lombardy method, and 0.90 for the anterior LUS score. As the output of the LUS methods for lung morphology classification is dichotomous, only one cut-off can be presented for the corresponding ROC curves. LUS, lung ultrasound; ROC, receiver operating characteristic.

The Amsterdam method performed significantly better than the Piedmont method [NRI: 0.179 (CI: 0.037–0.320), IDI: 0.179 (CI: 0.034–0.323), *p* = 0.015]. The Amsterdam method was not significantly better than the Lombardy method [NRI: 0.127 (CI: −0.063 to 0.318), IDI: 0.127 (CI: −0.067 to 0.322), *p* = 0.199]. There was no difference in classification between the Lombardy and Piedmont method [NRI: 0.051 (CI: −0.083 to 0.185), IDI: 0.051 (−0.086 to 0.188), *p* = 0.463]. ROC curves for the three LUS methods are presented in Figure 5.

For the Amsterdam method, 13 out of 18 patients in the Amsterdam cohort and 10 out of 10 patients in the Lombardy cohort could be classified as non-focal lung morphology solely based on the anterior LUS score. For the Lombardy method, 14 out of 14 patients in the Amsterdam cohort and in 7 out of 9 patients in the Lombardy cohort could be classified as focal lung morphology solely based on the anterior LUS score. Additional data on routes toward classification for the Amsterdam and Lombardy method is presented in Supplementary Results.

### Regional LUS Differences Between Morphologies

Anterior LUS scores were higher in patients with non-focal morphology compared to patients with focal morphology in both the Amsterdam [3 (1–5) vs. 0 (0–1), *p* < 0.001] and the Lombardy cohort [8 (5–8) vs. 1 (0–2), *p* < 0.001]. An ROC curve for the anterior LUS score is presented in Figure 5, showing that an anterior LUS score ≤2 is the most discriminant cut-off for classification of non-focal lung morphology in the Amsterdam and Lombardy cohort combined. The lateral LUS score was higher in patients with non-focal morphology compared to patients with focal morphology in the Amsterdam cohort [5 (3–7) vs. 1 (0–3), *p* = 0.012] but not in the Lombardy cohort [10 (8–12) vs. 10 (9–12), *p* = 0.803]. The posterior LUS score was not different between patients with non-focal morphology and patients with focal morphology in both the Amsterdam cohort [7 (5–9) vs. 4 (3–8), *p* = 0.166] and the Lombardy cohort [11 (10–11) vs. 12 (11–12), *p* = 0.054] (Figure 1).

## Discussion

The main findings of this study can be summarized as follows: (1) LUS-based methods can accurately classify lung morphology in invasively ventilated patients with ARDS, and (2) an anterior LUS score equal or larger than 2 was strongly related with a non-focal lung morphology.

Personalized ventilation based on lung morphology has great potential to improve treatment of individual ARDS patients, but only if lung morphology is correctly classified (Constantin et al., 2019). Chest CT is the gold standard for classification of lung morphology, but is commonly not feasible due to risky transport and CT can also be complex to interpret (Matthay et al., 2020). Therefore, there is a strong need for an accurate alternative to chest CT, that is available bedside and accessible for all ICU physicians. LUS can fill this implementation gap as LUS-based methods are objective and easy to apply in clinical practice as they rely on a well-defined and validated scoring system (Bouhemad et al., 2011). For example, previously implemented LUS methods with comparable complexity were reproducible between operators after limited training (Bouhemad et al., 2010; Mongodi et al., 2017; Rouby et al., 2018; Vercesi et al., 2018). Moreover, LUS is one of the tools that is also suitable for diagnosis and management of ARDS patients in limited resource settings (Riviello et al., 2016; Pisani et al., 2021).

Because there was uncertainty on the best approach toward estimating lung morphology with LUS we considered and studied several methods. The “AzuRea” group described a LUS method for assessment of lung morphology to evaluate changes in oxygenation following prone position (Haddam et al., 2016). However, this method did not capture lung morphology accurately and was not considered applicable to our population. Costamagna et al. proposed a LUS method based on anterior LUS scores for classification of lung morphology and validated the method with gold standard chest CT (Piedmont method) (Costamagna et al., 2021). This method performed excellent in the original study but the performance decreased substantially when applied to the Amsterdam and Lombardy cohorts. A possible explanation could be selection bias in the Piedmont study because only 23% of patients were classified as having focal lung morphology, which is substantially lower compared to other cohorts (Constantin et al., 2010, 2019). Another second reason could be the different approach in scoring B-patterns between the Piedmont study and the cohorts used in the present study.

The present study confirms that the anterior LUS scores are most important in classification of lung morphology. The fact that anterior LUS regions had the largest influence in classifying lung morphology enhances the applicability of LUS in clinical practice, as these regions are easy and quick to assess. As a misaligned ventilation strategy is probably worst for patients with focal lung morphology ventilated as a patient with non-focal lung morphology (Constantin et al., 2019), a low anterior LUS score could be an indication of low PEEP and prone position rather than alveolar recruitment maneuver. But although the anterior LUS score is most important, the posterior LUS score when compared to the lateral LUS score (Amsterdam method) or anterior LUS score (Lombardy method) should not be neglected. Incorporating these ratios in a two-step approach can significantly improve the performance of LUS methods and therefore avoid harmful misclassifications. Moreover, a complete 12-region LUS exam can be performed within 10 min by an experienced sonographer (Rouby et al., 2018).

The Amsterdam and Lombardy methods performed best when using data from the center they were derived from. Both methods had a high accuracy for lung morphology in their respected validation cohorts as well, with the Amsterdam method seemingly outperforming the Lombardy method. The major difference between these methods lies in the diagnostic approach: in the Amsterdam method a high anterior LUS score was used to confirm non-focal morphology whereas in Lombardy method a low anterior LUS score was used to confirm focal morphology. Both the Lombardy and Amsterdam method showed decreased performance during external validation. A possible explanation for this decrease is the significant difference in LUS scores between cohorts. The higher LUS scores in the Lombardy cohort might be the result of the lower PEEP settings or higher disease severity in this particular cohort. The original study where the Lombardy cohort was derived from showed that the global LUS score lowered with 4 points when PEEP was changed from 5 to 15 cmH_2_O (Chiumello et al., 2018). The difference in PEEP of 10 cmH_2_O in this previous study was, however, much larger than the difference in median PEEP of 3 cmH_2_O between the Amsterdam and Lombardy cohorts. It is therefore likely that the higher mortality and disease severity in the Lombardy cohort largely contributed to the higher LUS scores as well. Subsequently, patients with non-focal morphology and a low anterior LUS score were only found in the Amsterdam cohort where a higher clinically used PEEP was applied in patients with a low-moderate global LUS score. Patients with focal morphology and a high anterior LUS score were only identified in the Lombardy cohort where the global LUS score was higher and PEEP was fixed at 5 cmH_2_O per protocol.

The level of PEEP during assessment of lung morphology is important, as changes in PEEP alter lung aeration that is measured with lung imaging (Bouhemad et al., 2011; Corradi et al., 2016). It should be noted that previous studies assessed lung morphology at zero PEEP, but this was not done in both cohorts of this study (Constantin et al., 2010). This is unpractical, unethical and subsequent studies used a PEEP of 5 cmH_2_O (Constantin et al., 2019), which is in line with the PEEP used in the Lombardy cohort where patients were studied at PEEP of 5 cmH_2_O per protocol. Future studies should investigate at what PEEP level lung morphology should be assessed with LUS and then modify the LUS method accordingly.

This study has several strengths. The external validity of the study is high as we used two different cohorts of ARDS patients treated in different centers for development and validation of LUS-based methods for identification of lung morphology. LUS examination was identical in the two cohorts and the patients were examined almost simultaneously with CT examination. The validity of LUS to evaluate lung morphology was assessed at different levels of PEEP, with a varying level of PEEP in the Amsterdam cohort that reflects clinical practice in this institution. Nevertheless, this study also has several limitations. First, the validity of using the difference between lateral and posterior LUS scores in the Amsterdam method was not fully assessed as all the patients with non-focal morphology in the Lombardy cohort were classified solely based on the anterior LUS regions. Second, the sample size of both cohorts was small due to the limited availability of paired LUS and CT images using standardized protocols at the same PEEP settings. Therefore, prospective validation of the LUS methods is advised. Third, both cohorts did not include any patients with COVID-19 related ARDS, thus we cannot translate our findings to this prevalent disease.

In conclusion, LUS-based methods can accurately classify lung morphology in invasively ventilated ARDS patients compared to gold standard chest CT. The anterior LUS regions showed to be the most discriminant between focal and non-focal lung morphology, although accuracy increased moderately when lateral and posterior LUS regions were integrated in the method.

## Data Availability Statement

The original contributions presented in the study are included in the article/Supplementary Material, further inquiries can be directed to the corresponding author/s.

## Ethics Statement

The studies involving human participants were reviewed and approved by the Ethics Committees of the Academic Medical Center, Amsterdam, Netherlands (2017_312#B201859) and Fondazione IRCCS Cà Granda Ospedale Maggiore Policlinico, Milan, Italy. The patients/participants provided their written informed consent to participate in this study.

## Author Contributions

All authors contributed to study conception and design, and commented on previous versions of the manuscript. CP, MRS, FM, SM, and LDJB performed the analysis and wrote the first draft of the manuscript. All authors read and approved the final manuscript.

## Conflict of Interest

MJS reports personal fees from Hamilton and Xenios/NovaLung, outside of the submitted work. J-MC reports personal fees and non-financial support from Drager, GE Healthcare, Sedana Medical, Baxter, and Amomed, personal fees from Fisher and Paykel Healthcare, Orion, Philips Medical, and Fresenius Medical Care, and non-financial support from LFB, and Bird Corporation, outside of the present work. FM received fees for lectures from GE Healthcare, Hamilton Medical, SEDA SpA, outside the present work. A research agreement is active between University of Pavia and Hamilton Medical, outside the present work. SM received fees for lectures from GE Healthcare, outside the present work. LDJB reports grants from the Dutch lung foundation (Young investigator grant), grants from the Dutch lung foundation (Public–Private Partnership grant), grants from the Dutch lung foundation (Dirkje Postma Award), grants from IMI and from Amsterdam UMC, outside the submitted work. The remaining authors declare that the research was conducted in the absence of any commercial or financial relationships that could be construed as a potential conflict of interest.

## Publisher’s Note

All claims expressed in this article are solely those of the authors and do not necessarily represent those of their affiliated organizations, or those of the publisher, the editors and the reviewers. Any product that may be evaluated in this article, or claim that may be made by its manufacturer, is not guaranteed or endorsed by the publisher.

## Abbreviations

ARDS: acute respiratory distress syndrome
LUS: lung ultrasound
CT: computed tomography
ICU: intensive care unit
IRB: institutional review board
PEEP: positive end-expiratory pressure
IQRs: interquartile ranges
PPV: positive predictive value
NPV: negative predictive value
PaO_2_: partial pressure of oxygen
FiO_2_: fraction of inspired oxygen
ROC: receiver operating characteristic
NRI: net-reclassification index
IDI: integrated discrimination index.
