# Supplementary material for: Lung Ultrasound Assessment of Focal and Non-focal Lung Morphology in Patients With Acute Respiratory Distress Syndrome
Source: Front Physiol. 2021 Sep 14;12:730857. doi: 10.3389/fphys.2021.730857 (PMC8476947; doi:10.3389/fphys.2021.730857)

Supplementary Material

**Supplemental results**

Route towards classification using the Amsterdam and Lombardy methods

For the Amsterdam method, the majority of patients with non-focal lung morphology could be classified solely based on the anterior LUS score (13 out of 18 patients in the Amsterdam cohort and 10 out of 10 patients in the Lombardy cohort). In patients from the Amsterdam cohort with non-focal lung morphology that were not classified based on the anterior LUS score, 3 out of 5 patients were correctly classified based on a lateral LUS score that was higher than the posterior LUS score in at least one hemithorax. The other two patients with non-focal lung morphology from the Amsterdam cohort could not be classified based on the anterior LUS score or the ratio between lateral and posterior LUS scores, and were misclassified. In the Lombardy cohort, two patients with focal lung morphology were misclassified as having non-focal lung morphology by the Amsterdam method due to an anterior LUS score ≥ 2.

For the Lombardy method, most patients with focal lung morphology were correctly classified solely based on anterior LUS scores (14 out of 14 patients in the Amsterdam cohort and in 7 out of 9 patients in the Lombardy cohort). Using the Lombardy method, 7 out of 18 patients in the Amsterdam cohort with non-focal lung morphology were misclassified as having focal lung morphology, all explained by the presence of an anterior LUS score ≤ 2. In patients from the Lombardy cohort, 1 out of 10 patients with non-focal lung morphology was misclassified by the Lombardy method because the posterior LUS score was higher than two times the anterior LUS score.

**Supplemental figures**

**Figure 1**. Examples of ARDS lung morphology categorized as ‘focal’ or ‘non focal: a) Consolidations isolated in the dorsal areas (focal morphology), b) Diffuse patchy loss in ventral and/or dorsal areas without any consolidations (non-focal morphology), c) Consolidations in the dorsal areas and diffuse patchy loss in ventral areas (non-focal morphology), d) Consolidations in one lung at dorsal areas and diffuse patchy loss with or without consolidation in the other lung (non-focal morphology)


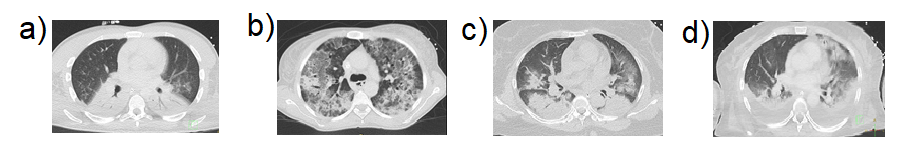


**Figure 2.** Lung ultrasound image of a consolidated lung ultrasound pattern using a phased array transducer.


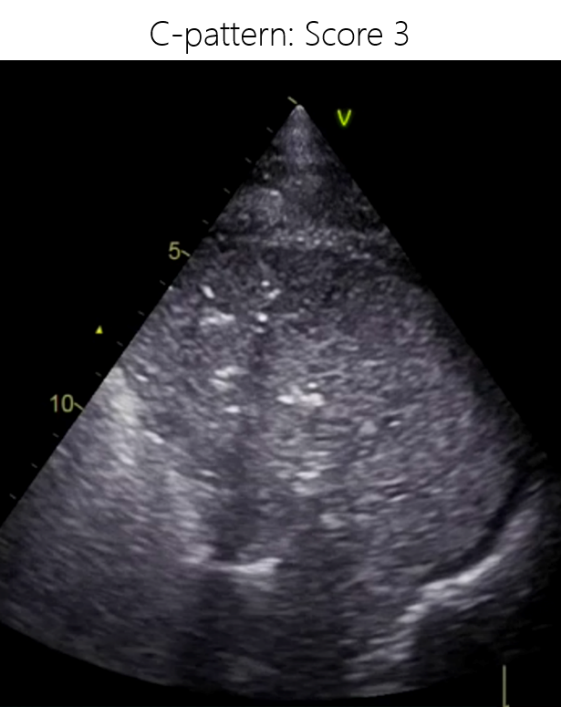

Supplement: Supplementary file 7 [file Data_Sheet_1.DOCX]
